# Social and Cultural Sustainability: Criteria, Indicators, Verifier Variables for Measurement and Maps for Visualization to Support Planning

**DOI:** 10.1007/s13280-012-0376-0

**Published:** 2013-03-10

**Authors:** Robert Axelsson, Per Angelstam, Erik Degerman, Sara Teitelbaum, Kjell Andersson, Marine Elbakidze, Marcus K. Drotz

**Affiliations:** 1 Faculty of Forest Sciences, School for Forest Management, Swedish University of Agricultural Sciences, PO Box 43, 739 21 Skinnskatteberg, Sweden; 2 Faculty of Forest Sciences, School for Forest Management, Swedish University of Agricultural Sciences, PO Box 43, 730 91 Skinnskatteberg, Sweden; 3 Faculty of Aquatic Resources, Institute of Freshwater Research, Swedish University of Agricultural Sciences, 702 15 Örebro, Sweden; 4 Université du Québec à Montréal, P.O. Box 888, Centre-ville Station, Montreal, QC H3C 3P8 Canada; 5 The Lake Vänern Museum of Natural and Cultural History [Vänermuseet], Framnäsvägen 2, 531 54 Lidköping, Sweden

**Keywords:** Municipality, Policy implementation, Sustainable development, Sweden

## Abstract

Policies on economic use of natural resources require considerations to social and cultural values. In order to make those concrete in a planning context, this paper aims to interpret social and cultural criteria, identify indicators, match these with verifier variables and visualize them on maps. Indicators were selected from a review of scholarly work and natural resource policies, and then matched with verifier variables available for Sweden’s 290 municipalities. Maps of the spatial distribution of four social and four cultural verifier variables were then produced. Consideration of social and cultural values in the studied natural resource use sectors was limited. The spatial distribution of the verifier variables exhibited a general divide between northwest and south Sweden, and regional rural and urban areas. We conclude that it is possible to identify indicators and match them with verifier variables to support inclusion of social and cultural values in planning.

## Introduction

Policies and guidelines about the sustainable use of natural resources encompass not only ecological and economic, but also social and cultural dimensions (Throsby 1999; Council of Europe 2000; Hawkes 2001; Littig and Grießler 2005; Forest Europe, UNECE and FAO 2011). While the social dimension together with the ecological and economic are well established parts of the sustainable development concept (WCED 1987), there is ongoing debate about the need to include a fourth, the cultural dimension (Saastamoinen 2005; UNESCO 2010; Culture 21 2011; Chan et al. 2012; Daniel et al. 2012). The need to increase understanding and methodological development related to social and cultural values in planning is explicitly emphasized in the European Landscape Convention (ELC) (Council of Europe 2000), and is also addressed in sector-specific policies (e.g., MCPFE 1993; WFD 2000; Forest Europe, UNECE and FAO 2011). According to the ELC, all signatory states should define landscapes, assess their qualities, and form policy about them. In addition the signatory states should establish collaboration amongst all sectors and actors representing different interests to facilitate planning for sustainable landscape protection and management decisions (Council of Europe 2000; Uzun and Müderrisglu 2011).

For a given landscape understood as a space and a place (sensu Grodzynskyi 2005), social and cultural criteria encompass objects and structures, such as historical remains and habitat for people, and values such as sense of place, local culture, and traditions (Fairclough and Rippon 2002; Antrop 2003; Palang and Fry 2003; Claval 2004). To provide transparent information for decision-makers and stakeholders about the state and trends of social and cultural sustainability, data on both material and immaterial landscape values are required (Vos and Meekes 1999; Termorshuizen and Opdam 2009; Angelstam et al. 2013a). However, these social and cultural dimensions are not easy to define or measure, and their inclusion in planning is not well developed (Colantonio 2007; Magis and Shinn 2009). Consequently, there is a need to interpret policy and practice from different landscape contexts, to choose suitable indicators (sensu Lammerts van Bueren and Blom 1997) and basic methods for monitoring (Antonson et al. 2010; Mikusiński et al. 2012). Defining and measuring verifier variables can inform planning decisions with the status of indicators and shed light on the impacts of these decisions. Additionally, effective means of visualization and communication using maps are needed in order to facilitate learning and understanding of the status and trends of social and cultural sustainability (Lee 1993; Curtis 2004; Hajkowicz and Collins 2007, Bell and Morse 2008; Marinoni et al. 2009; Zetterberg 2009; Andersson et al. 2012).

Sweden is a country with a long history of natural resource use (Antonson and Jansson 2011) that has been important for the country’s development towards a modern industrial society (Sörlin 1988; Schön 2011). The historical development of business sectors using different natural resources initially focused on economic development (Heckscher 1968). This is still the case today, even if considerations to other sustainability dimensions have emerged (Lehtinen et al. 2004; Chen and Jonson 2008; Angelstam et al. 2011). Since the first UN Conference on Environment, held in Stockholm in 1972 and the subsequent emergence of the sustainable development (SD) discourse (WCED 1987), Sweden has put a great deal of effort towards SD (Rowe and Fudge 2003). In fact, Sweden has worked hard to integrate social and environmental concerns into its work with SD (Schön 2011). This makes Sweden an interesting case to study with respect to the state of social and cultural sustainability in its different regions.

The aim of this study is to present an approach to make social and cultural values concrete in a planning context. We interpreted social and cultural criteria by reviewing literature, policies and six natural resource management sectors, identified a set of social and cultural indicators, matched these with verifier variables, and visualized on maps for all Swedish municipalities.

## Methodology

The analysis was based on the ladder from criteria (principle or standard), indicators (indicating the sustainability status) to verifier variables (a monitored value that provides data for an indicator) of sustainability proposed by Lammerts van Bueren and Blom (1997). Indicators for sustainability are often used together with norms (thresholds or target values) defined in policies to assess the degree of sustainability. In this study this final step is not included.

First, we reviewed international policies and scholarly work to present a brief interpretation of social and cultural sustainability criteria. Multiple methods were used. Using the search words social and cultural we searched for relevant policy document and scholarly work. This was complemented by contacts with people and researchers, found as a part of the review, in this field that recommended additional literature.

Second, based on a review of scholarly work, and policies we identified a set of indicators (sensu Lammerts van Bueren and Blom 1997) which could be applied in a planning context. This included a review of six natural resource use sectors in Sweden, in order to understand how each considers social and cultural values. The sectors included the four main natural resource sectors in Sweden, i.e., agriculture, forestry, hydro-power, and mining. In addition we included wind energy, and the process of land consolidation. This is a process where the Swedish mapping, cadastral and land registration authority works with land owners, supported by a regional administration and the Swedish Forest Agency to produce a less fragmented land ownership pattern to allow a more efficient use of natural resources. Using expert interviews (sensu Flick 2006) we determined whether policies considered social and cultural values, and if these were considered in practice for the selected sectors. The interviews were short and concise (ca. 10 min). We contacted and interviewed at least one natural resource use representative of each sector. This was either a person working for a government agency or a company. The informants were selected by asking for someone with knowledge about social and cultural values. It often took several calls to identify a person with the sought after expert knowledge. In addition, a person from a non-government environmental organization was interviewed. These were selected in a similar way. Data were collected by filling out a table with references to mentioned policies and/or a notice about practices in a table. When the dataset was considered saturated we had interviewed a total of 30 persons (Kvale 2007).

Third, we matched the selected indicators with freely available official data as verifier variables. The only exception was data on forest cover in urban areas that was a part of a commercially available dataset from the Swedish mapping, cadastral and land registration authority.

Fourth, we made maps for all Sweden’s 290 municipalities. In addition, we analyzed relationships among the verifier variables, which were not normally distributed, using Spearman rank correlation. This was done in order to determine if variables were highly correlated, indicating that one variable may be redundant. We also studied the correlation between verifier variables and climate, population size, education level, number of universities, and the average income. This was done to see which potential drivers were correlated with the indicators, thereby giving explanation to the distribution of indicators on the maps produced. Population size was expressed in total numbers and as inhabitants per km^2^. As a proxy for climate the so-called normal temperature, or annual average air temperature for a 30-year period (1961–1990) was used (SMHI 2012). The education level was given as percent people with university education.

### Sweden as a Case Study

Sweden is divided into 21 regional administrations or counties and 290 local municipalities. Municipalities are to a large degree independent from the state. Inhabitants elect a local parliament as a part of national elections every 4th year. Municipalities are responsible for large parts of social services such as schools, libraries, child care, emergency services, elderly care, and social service, while the regional administrations handle issues like health care, culture, and public transport. The government and the parliament decide about policies, and government agencies support the implementation of those policies. Municipalities have full responsibility for comprehensive planning of their territory, while regional administrations and government agencies can produce plans of an advisory character. The county administration, as the regional representative of the government, ensures that municipal plans are in line with laws, regulations and policies. Permits for use of natural resources might include decisions from different government agencies, regional administrations, and municipalities.

## Results

### Interpretation of Social and Cultural Sustainability Criteria

In this first step we review and interpret social and cultural sustainability criteria. According to the Merriam-Webster dictionary (Merriam Webster 2012) the term *social* relates to “human society, the interaction of the individual and the group, or the welfare of human beings as members of society”. Social sustainability was originally introduced as a part of the SD concept in the Brundtland report (WCED 1987). The main definition of SD, namely “development which meets the needs of the present without comprising the ability for future generations to meet their own needs” has a clear social imperative. The Brundtland report focused on issues like health, and the income gap between rich and poor with an aim to reduce poverty globally. The Rio conference in 1992 introduced social sustainability as the right to live a decent life; inter-generational, intra-generational, and international social justice; and local participation in SD processes. This was further elaborated by including issues like welfare, safety and a healthy environment, access to education, opportunities to learn, identity, sense of place, and public participation. The concept of social sustainability continues to develop. Thin (2002) describes social justice, solidarity, participation, and security as social values. Social values can be characterized as conditions associated with quality of life in the landscape, including such things as equity, participation in democratic life, security and health (Rosenström et al. 2006). Recent additions include concepts like human well-being, happiness, and quality of life (Colantonio 2007; Table 1). For a more comprehensive review of social sustainability, see Murphy (2012).

**Table 1:** Social and cultural criteria defined in early conventions (UNESCO 1972, 2003), new themes from international policies and scholarly work (compiled from Council of Europe 2000; Saastamoinen 2005; Colantonio 2007) and emerging from the Rio+20 process (Culture 21 2011)

	Cultural sustainability	Social sustainability
Material	Early: Cultural heritage in terms of human built objects, landscapes and combined man and nature systems	–
Immaterial	New: Cultural heritage such as in terms of practices, representations, expressions, knowledge, skills, and instruments, objects, artefacts and cultural spaces associated with practices, including tradition, identity, values, cultural diversity, spirituality, and esthetics	Traditional: Welfare, housing and environmental health Education and skills Employment Equity Human rights and gender Poverty Social justice
	Emerging: Tools and skills needed to understand and transform the world towards sustainability, including but not limited to literacy, creativity, critical knowledge, sense of place, empathy, trust, risk, respect, and recognition	Emerging: Demographic change (aging, migration, mobility) Social integration and cohesion Identity, sense of place and access Health and safety Social capital Wellbeing, happiness and quality of life

There are many definitions of *culture* (Kroeber and Kluckhohn 1952). To select one, culture could be described as: (1) the mind of a cultured person; (2) the process of culturing people; (3) art and intellectual works that might culture a person; and (4) culture as a system that maintains, communicates, and reproduce the characteristics of a society, and that allows for people to participate in it (Williams 1981). Cultural sustainability was first mentioned in 1995, when the World Commission on Culture and Development (WCCD), building on the SD discourse, defined cultural sustainability as inter- and intra-generational access to cultural resources (WCCD 1995). Cultural heritage is defined as “the entire corpus of material signs - either artistic or symbolic - handed on by the past to each culture and, therefore, to the whole of humankind” (UNESCO 1989). Tangible parts include monuments of architectural, sculptural, painted, and archeological nature and human-made landscapes (UNESCO 1972). While intangible cultural heritage include “practices, representations, expressions, knowledge, skills – as well as the instruments, objects, artefacts and cultural spaces associated therewith – that communities, groups and, in some cases, individuals recognize as part of their cultural heritage.” (UNESCO 2003). In 2001 a process with the aim to add culture as the fourth sustainability dimension started with the UNESCO Universal Declaration on cultural diversity (UNESCO 2001), which is also argued for by scholars (e.g., Saastamoinen 2005). This argumentation has continued with the Rio+20 process (UN 2012). The cultural working group under the Rio+20 process describes the present situation as “Today human beings have the capacities but do not have some of the capabilities (tools and skills) to understand the world and to transform it so that it becomes really sustainable”. Capabilities such as literacy, creativity, critical knowledge, sense of place, empathy, trust, risk, respect, recognition, to list a few, could then be understood as cultural components of sustainability (Culture 21 2011).

For both social and cultural sustainability there is an ongoing development from a more traditional view that focuses on material cultural heritage and basic social needs to a view including also immaterial aspects (Table 1). These encompass both tangible and intangible values.

### Indicators for Social and Cultural Values

To illustrate the second step we identified four social and four cultural indicators based on our review of scholarly work, international policies, and six natural resource use sectors’ policies (Table 2). The review and the expert interviews related to individual natural resource sectors showed that these policies rarely considered more than one or two of the indicators. In addition, considerations directed towards these were rarely well developed and supported by scholarly work (Table 2). There were no traces of other social or cultural indicators in sector policy or practice. This is surprising given that the importance of integrating social and cultural values in landscape planning is widely recognized at the international policy level.

**Table 2:** Selected indicators of social and cultural sustainability criteria from international policies, scholarly work and individual natural resource use sectors

	Indicator	Scholarly references	General policies	Appearance in sector policies and practice
Social sustainability	Democratic civil society	Rothstein and Uslaner (2005), Magis and Shinn (2009)	UN (1998), Hantrais (2007)	Wind energy^a^, land consolidation^a^ (improved local processes)
	Living environment	Magis and Shinn (2009), Grahn and Stigsdotter (2010)	EU (2010, 2011)	Forestry (Swedish Forest Agency 2011a, b)
	Human development	Magis and Shinn (2009)	UNDP (2007)	Mining^a^ (development of sustainable mining)
	Equity	Rothstein and Uslaner (2005), Magis and Shinn (2009)	UNDP (2007)	
Cultural sustainability	Cultural vitality, diversity and conviviality, Social capital	Putnam (2000), Mercer (2002), Magis and Shinn (2009)	RAA (Swedish National Heritage Board) (2005)	Wind energy^a^, hydropower^a^ (support to local NGOs)
	Cultural landscape	Vos and Meekes (1999), Oñate et al. (2000), Nohl (2001), Palang and Fry (2003)	Föreningen Sveriges Länsantikvarier (2004), Council of Europe (2000), Van Mansvelt and Van der Lubbe (1998)	Agriculture (Landsbygdsdepartementet 2012)
	Cultural heritage	Palang and Fry (2003)	Council of Europe (2000), Jakobsson et al. (2010), SOU (2012)	Forestry (Swedish Forest Agency 2011c), Agriculture (Landsbygdsdepartementet 2012)
	Cultural access, participation, consumption	Mercer (2002)	RAA (Swedish National Heritage Board) (2005)	

^a^Weaker occurrences that are not established as policies

While measurements of social and cultural landscape values presents methodological challenges (Scazzosi 2004; Tress et al. 2006; Naveh 2007), a set of concepts with accompanying measurements can be discerned which are being used by scholars and policy makers to identify both social and cultural values at a landscape level. Following the comprehensive review by Magis and Shinn (2009), we interpret social values as four groups of indicators:

1. Democratic civil society, including participation in the development process locally. The transition process from government to governance is an important part of this indicator and a prerequisite for further democratic development in many societies.
2. Living environment, which include human wellbeing and safety related to natural disasters and social unrest, the need to understand esthetic values, health preferences and health effects of populations towards the environment. There has been considerable research done about human perceptions of different landscapes and landscape features using a variety of methods including surveys, photo-based studies, and in-depth interviews (Herzog 1987; Gyllin and Grahn 2005; Grahn and Stigsdotter 2010). In addition, some studies have shown direct links between the landscape and human health (de Jong et al. 2012; for a review, see Tzoulas et al. 2007).
3. Human development related to health, education, income and potentially other parameters. There are several indicator and index frameworks designed to provide information on quality of life, complete with statistical measures, at international, national, and local levels (UN 1996, 2007; Bartelmus 1997; Bell and Morse 2008; Carraro et al. 2009).
4. Equity as equal rights, opportunities, education, income, and health (Uslaner 1999; Rothstein and Uslaner 2005; Table 2).

Applied research on cultural values is most often associated with cultural heritage objects and cultural landscapes, described as the “bearers of the place identity, or *genius loci*” (Dramstad et al. 2001; Aluame et al. 2003). While culture heritage objects have been identified for centuries, the cultural landscape is a more recent concept, based on the desire to treat the “entire landscape as an artefact” (Sauer 1925; Scazzosi 2004). The identification and evaluation of cultural landscapes is inherently more complex than of cultural heritage objects. As criteria to describe cultural landscapes, Antrop (2003) proposed historical significance (coherence, information from the past), esthetic qualities (naturalness, authenticity, stewardship), and utility (accessibility, monetary value). Similarly, there are other cultural values on the landscape that derive their value from contemporary uses such as recreation activities (UK Forestry Commission 2002; Sheppard et al. 2005) and self-provisioning activities, such as the harvesting of fish, berries, wild mushrooms, and wildlife (Crone and Haynes 2001). Finally, it is also possible to characterize cultural values as a type of ‘capital’ present on a given territory. Bourdieu (1986) described cultural capital as present in three forms: (1) the embodied state, i.e., as long-lasting dispositions of the human mind and body; (2) the objectified state, i.e., as any kind of cultural goods, such as pictures, books, instruments, machines; and (3) other human built objects such as buildings and structures, the institutionalized state or academic and educational qualifications. The subsequent use of the term is somewhat confusing since scholars often select one of the three forms and use it as if it represents the whole concept (Kraaykamp and van Eijck 2010). By contrast, Bourdieu (1986) defined social capital as social obligations or connections. Similarly, Putnam (2000) described social capital as the social networks and norms that enable collective action, emphasizing the importance of social capital for the creation of a vibrant democratic system. Inventorying and mapping the cultural policy environment (presence of cultural institutions, level of participation in cultural life, etc.) is one way to illustrate this dimension of social capital. Hence, social capital is a cultural property of a human community. Mercer (2002) describes four categories which can be used to monitor the success of cultural policies for human development. These are: (a) cultural vitality, diversity, and conviviality; (b) cultural access, participation, and consumption; (c) culture, lifestyle, and identity; and (d) culture, ethics, governance, and conduct. The society of Swedish regional heritage officers identified 16 indicators for cultural values, emphasized three and pointed out one, the number of active farms as the most important (Föreningen Sveriges Länsantikvarier 2004). We selected (1) cultural vitality, diversity, and conviviality or social capital; (2) cultural landscape; (3) cultural heritage; and (4) cultural access, participation, and consumption as cultural indicators (Table 2).

To conclude, while the term social relates to the individual, family, or individuals in a society, the term cultural relates to higher societal levels, i.e., properties of groups of people, communities, and regions or systems (White 1975). This means that “cultural” relates to a non-biological system of development and adaptation (Steward 1955). Culture thus includes any kind of heritage from the past, ranging from how people interact and do things to any kind of object, or environments that are a results of human constructions or use of the landscape. Recognizing that social and cultural criteria to some extent overlap, we nevertheless identified two groups of indicators for further analysis and to demonstrate this approach.

### Verifier Variables for the Selected Indicators

The third step in our approach was to match the indicators with available data as verifier variables (Table 3). Data available at a municipal level that best matched the indicators were used. Indicators for human development and gender equity were based on the Human Development and Gender Development Indexes (HDI and GDI) (UNDP 2007). To use these indexes for all Swedish municipalities we simplified them by using variables for health, i.e., life expectancy at birth (FHI 2011), average income (Statistics Sweden 2010), higher education among people 25–64 years old (Statistics Sweden 2011b) and followed the careful instruction in UNDP (2007). While the HDI presents a measure of human development, the GDI compares the situation for men and women. To avoid confusion with the Human Development and Gender Development Indexes we named these verifier variables Index of Human Development (IHD) and Index of Gender Development (IGD).

**Table 3:** Indicators (see Table 2), verifier variables with units, and data sources for social and cultural sustainability criteria (see Lammerts van Bueren and Blom 1997 for terminology)

Indicator	Verifier/variable (unit)	Data sources
Democratic civil society	Participation in local elections (%)	Swedish Election Authority (2010)
Living environment	Forest in urban areas (%)	Swedish mapping, cadastral and land registration authority (2011)
Human development	Index of Human Development (UNDP 2007; using data on health, education and income) (index value 0–100)	FHI (Swedish National Institute of Public Health) (2011), Statistics Sweden (2010, 2011b)
Equity	Index of Gender Development (UNDP 2007; using data on health, education and income) (index value 0–100)	FHI (Swedish National Institute of Public Health) (2011), Statistics Sweden (2010, 2011b)
Cultural vitality, diversity and conviviality, Social capital	Number of voluntary groups (*n*/1000 inhabitants)	Statistics Sweden (2011a, 2012b)
Cultural landscape	Number of active farmers (*n*/km^2^)	Swedish Board of Agriculture (2011), Statistics Sweden (2012a)
Cultural heritage	Historical remains (*n*/km^2^)	RAA (Swedish National Heritage Board) (2012), Statistics Sweden (2012a)
Cultural access, participation and consumption	Number of available cinemas showrooms, theaters, museums and libraries (*n*/municipality)	National Library of Sweden (2011), Swedish Arts Council (2009), Swedish Film Institute (2011), Statistics Sweden (2012c)

### Visualization of Data as Maps for Swedish Municipalities

**Fig. 1:**
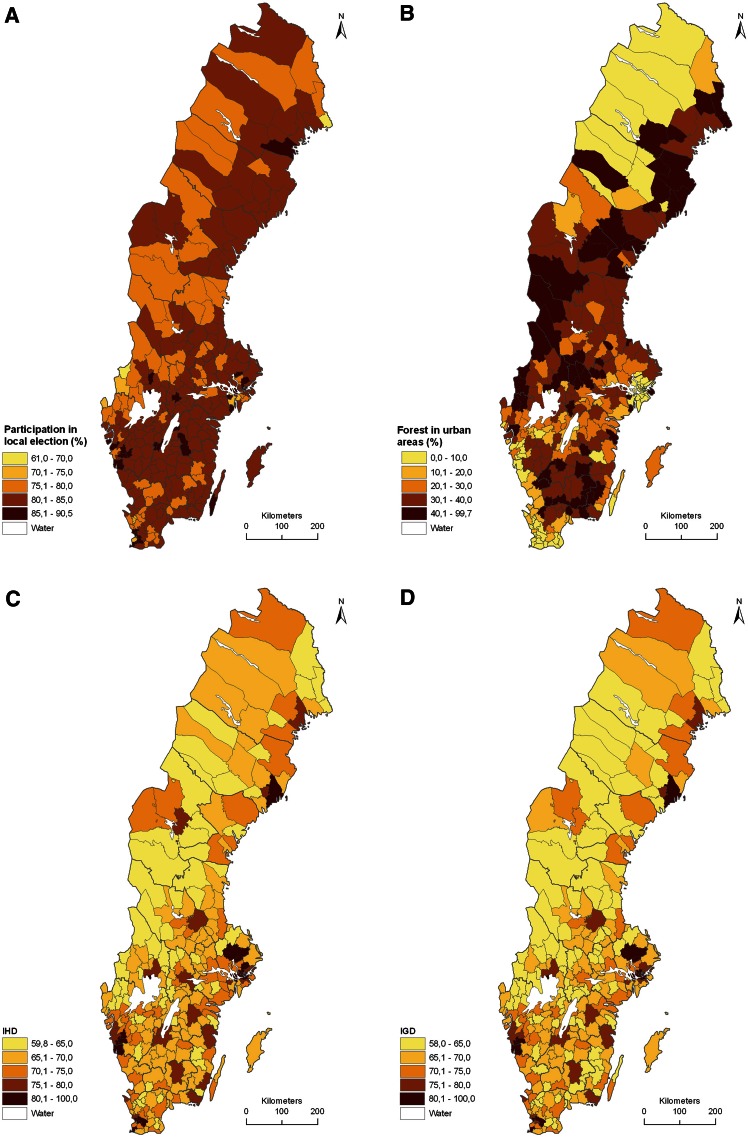
Maps of parameter values for four verifier variables for social sustainability in Sweden’s 290 municipalities: **A** participation in local election (%), **B** forest in urban areas (%), **C** Index of Human Development (IHD), and **D** Index of Gender Development (IGD). For details see Table 3

**Fig. 2:**
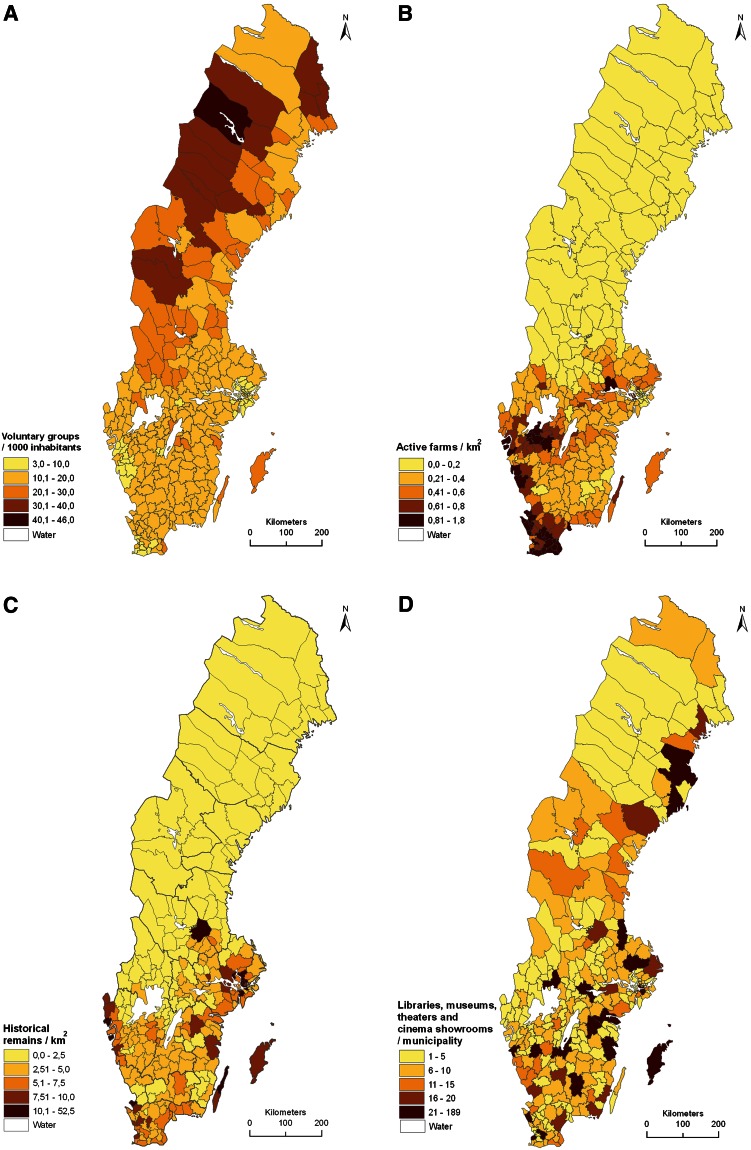
Maps of parameter values for four verifier variables for cultural sustainability in Sweden’s 290 municipalities: **A** voluntary groups/1000 inhabitants, **B** active farms/km^2^, **C** historical remains/km^2^ land area, and **D** Sum of libraries, museums, theaters, and cinema showrooms/municipality. For details see Table 3

The fourth step was to visualize the verifier variables as maps. There was a general divide in the spatial variation of verifier variables for social (Fig. 1) and cultural (Fig. 2) sustainability between northwest and south Sweden as well as between urban and rural regions. However, for participation in local elections there was no clear pattern. Rural region municipalities in general had a higher percentage of urban forest than urban regions. IHD and IGD scored higher for some municipalities with large cities and universities while social capital was higher in the north and especially the northwest. The number of active farms per km^2^ was higher in the south, below the ecological boundary separating the temperate deciduous and the boreal forest eco-regions in Sweden. Historical remains had a similar pattern except for the municipality of Falun in the Bergslagen region, that hosts a large historical mining site (Angelstam et al. 2013b) and many related historical remains. Also the verifier variable available art was higher in the south, included some municipalities with high values along the northern east coast and some scattered municipalities with fairly high values in mid-Sweden.

Of the chosen social and cultural verifier variables IHD and IGD were highly correlated (Table 4). This suggests that of these two social indicators one was redundant in the present data set. It should be stressed that among the cultural indicators “Voluntary groups per 1000 inhabitants” was negatively correlated to other cultural indicators, indicating another direction of the overall gradients. Top-ranked municipalities for this indicator were mainly small municipalities in the inland of northern Sweden, whereas cultural landscape (as number of active farmers/km^2^), heritage (as historical remains/km^2^), and access (as number of available cinemas showrooms, theaters, museums, and libraries/municipality) had the highest ranks for southern and densely populated areas.

**Table 4:** Spearman rank bivariate correlation between social and cultural verifier variables. Significant values (*P* < 0.05) using two-tailed tests are marked with an asterisk (*n* = 290)

	Forest proportion	IHD	IGD	Voluntary groups/1000 inhabitants	Active farms/km^2^	Historical remains/km^2^	Available art/municipality
Voter proportion	−0.160*	0.605*	0.600*	−0.339*	0.161*	0.243*	−0.020
Forest proportion		−0.446*	−0.442*	0.391*	−0.274*	−0.385*	−0.071
IHD			0.997*	−0.568*	0.178*	0.505*	0.371*
IGD				−0.585*	0.195*	0.523*	0.382*
Voluntary groups/1000 inhabitants					−0.417*	−0.594*	−0.031
Active farms/km^2^						0.550*	0.080
Historical remains/km^2^							0.186*

The proportion of the population with a higher education, and the average income, were important factors for computing IHD and IGD. In fact, these two factors were highly correlated with all social indicators, whereas climate (average air temperature) was not (Table 5). As for cultural indicators, climate (average air temperature) was an important factor with a high correlation to especially “Active farms per km^2^” and “Historical remains per km^2^”. Also population density was generally highly correlated to cultural indicators. To conclude, important drivers that were correlated to social indicators were income and education, and for cultural indicators climate and population density.

**Table 5:** Spearman rank bivariate correlation of social and cultural indicators versus external factors. Significant values (*P* < 0.05, *n* = 290) are marked with an asterisk. The normal temperature from 1961 to 1990 was used as a proxy for climate (SMHI 2012). Data on universities is from the Swedish National Agency for Higher Education’s yearly report 2011 (HSV 2011). For sources to all other datasets used see Table 3

Verifier variable	External factors					
	Climate	Population	Population/km^2^	Education	Universities	Av. income
Voter proportion	0.180*	0.207*	0.267*	0.527*	0.100	0.623*
Forest proportion	−0.323*	−0.322*	−0.439*	−0.445*	−0.008	−0.355*
IHD	0.268*	0.710*	0.631*	0.956*	0.362*	0.857*
IGD	0.296*	0.723*	0.654*	0.955*	0.365*	0.850*
Voluntary groups/1000 inhabitants	−0.586*	−0.462*	−0.806*	−0.470*	0.050	−0.618*
Active farms/km^2^	0.723*	0.255*	0.475*	0.184*	0.025	0.069
Historical remains/km^2^	0.665*	0.463*	0.689*	0.468*	0.053	0.427*
Available art/municipality	0.116	0.701	0.211*	0.425*	0.513*	0.179*

## Discussion

### Spatial Patterns of Social and Cultural Verifier Variables

The social and cultural verifier variables indicated a divide between south and northwest Sweden, and between larger urban centers such as regional capitals and cities with universities on the one hand, and rural areas on the other. The exceptions were the indicator for social capital as described by the verifier variable voluntary groups per 1000 inhabitants (which scored the highest in the western part of northern Sweden), and green infrastructure in urban areas (with higher scores in northwest and in rural areas). Important drivers for social indicators were income and education, and for cultural indicators climate and population size. Our approach shows that it is possible to use verifier variables for visualizing different sustainability indicators. This approach can be used as a base-line for learning and implementing sustainability policy and to support, for example, rural development.

Several studies indicate the importance of social and cultural values for economic development (Knack and Keefer 1997; Florida 2012), rural development (Van der Ploeg et al. 2000; Sorensen 2009), and human health (Grahn and Stigsdotter 2010). This should be of great interest for areas with a declining economy and population that has been pointed out as vulnerable (Tillväxtverket 2011) such as Bergslagen in Sweden (Angelstam et al. 2013b). To steer SD as a whole including social, economic, ecological, and cultural dimensions of sustainability requires knowledge about relevant verifier variables concerning the status and trends of all the four sustainability dimensions. In addition, a SD process based on collaborative learning processes among stakeholders is needed (Lee 1993). Some scholars have called this adaptive governance (Folke et al. 2005). A prerequisite for planning towards sustainability is that planners and decision-makers understand the terminology, the policy and can interpret the ambition of the policy as a certain target level (Dovers and Lindenmayer 1997; Van Herten and Gunning-Schepers 2000; Angelstam et al. 2004). A next step for this research will be the initiation of a learning process with stakeholders, to offer it to planners and decision-makers with the aim to produce socially robust knowledge (Nowotny 1999). This process could potentially follow the Aristotle model for sustainable knowledge as interpreted by Gustavsson (2000), where researchers’ scientific results need to be integrated with practical/experiential and political knowledge (as expressed in policies) to form socially robust knowledge.

### Inclusion of Social and Cultural Values is Less Developed

As the aim of this paper is to present a new approach for demonstrating utility, visualizing social and cultural values in a planning context and as more work remains to be done with indicators and especially verifier variables, we will not discuss specific results further. Instead the discussion will from now on focus on the challenge of social and cultural sustainability related to our approach.

Social and cultural criteria described in high-level policy are often not being implemented at a local level, i.e., there is a so-called disconnect (Dramstad et al. 2001; Bastian 2002; Scazzosi 2004). The datasets used as verifier variables for social and cultural sustainability were all a part of official and freely available datasets in Sweden. Nevertheless, still many of them are not actively used to support political steering and to provide information on the status and trends of sustainability locally (e.g., Andersson et al. 2012).

Our review of six natural resource use sectors showed that the inclusion of social and cultural values in their policy and practice was weak (see Table 2). To explain this we hypothesize that natural resource use policies have traditionally focused on economic dimensions. While ecological sustainability has a longer history of inclusion, social and especially cultural sustainability are more recent (Dillard et al. 2009). At international or general levels policies thus include all these dimensions of sustainability. However, the review emphasized that social and cultural sustainability have not yet been implemented or have not yet reached the practical level where they may impact landscape planning directly. Thus, it is clear that both social and cultural sustainability dimensions lag behind economic, but also the ecological, dimension. In addition, the inclusion of social and cultural values in natural resource management and planning requires both improved knowledge and a collaborative learning process among stakeholders (Bouwen and Taillieu 2004).

### Opportunities and Challenges of Assessing Social and Cultural Values

We made an attempt to synthesize ideas and efforts to measure social and cultural sustainability at the municipal level by integrating results from international policies, scholarly work, sector-specific policies, and practice. However, our interviews showed that policies for six natural resource use sectors were not well developed regarding social and cultural values. This is in line with the conclusions of Patterson and Williams (1998) and Dovers (2003).

This study demonstrates that it is possible to operationalize these concepts using available official data at the level of municipalities. Nevertheless, our approach is not unambiguous. An example is the verifier variable used to indicate social capital, i.e., voluntary groups per 1000 inhabitants, which is based on Putnam (2000). There is some evidence that trust and equity would be more appropriate verifier variables for social capital (Rothstein and Uslaner 2005) and especially in relation to economic development (Knack and Keefer 1997). In addition, the civic sector in Sweden is changing its structure (Lundström and Svedberg 2003), and social media seem to be a factor that needs to be considered (Ellison et al. 2007). Still, no available data captured social capital better than voluntary groups.

We conclude that it is possible to identify indicators and match them with verifier variables to support inclusion of social and cultural values in planning. There is, however, more work to do when it comes to the selection of indicators and verifier variables. To make this approach operational there is also a need for the final step, to identify target levels, such as expressed in policies. This would then allow for social and cultural sustainability assessments. We argue that the use of maps to visualize the sustainability status will assist stakeholders in the process of defining indicators, verifier variables, and target levels.
